# An Invasive Ductal Carcinomas Breast Cancer Grade Classification Using an Ensemble of Convolutional Neural Networks

**DOI:** 10.3390/diagnostics13111977

**Published:** 2023-06-05

**Authors:** Eelandula Kumaraswamy, Sumit Kumar, Manoj Sharma

**Affiliations:** 1 School of Electronics and Electrical Engineering, Lovely Professional University, Phagwara 144411, Punjab, India; kumaraswamy441@gmail.com; 2 Division of Research & Development, Lovely Professional University, Phagwara 144411, Punjab, India; 3 Department of ECE, Giani Zail Singh Campus College of Engineering & Technology, MRSPTU, Bathinda 151001, Punjab, India

**Keywords:** histopathological images, breast cancer, convolutional neural networks, computer-aided design, ensemble model

## Abstract

Invasive Ductal Carcinoma Breast Cancer (IDC-BC) is the most common type of cancer and its asymptomatic nature has led to an increased mortality rate globally. Advancements in artificial intelligence and machine learning have revolutionized the medical field with the development of AI-enabled computer-aided diagnosis (CAD) systems, which help in determining diseases at an early stage. CAD systems assist pathologists in their decision-making process to produce more reliable outcomes in order to treat patients well. In this work, the potential of pre-trained convolutional neural networks (CNNs) (i.e., EfficientNetV2L, ResNet152V2, DenseNet201), singly or as an ensemble, was thoroughly explored. The performances of these models were evaluated for IDC-BC grade classification using the DataBiox dataset. Data augmentation was used to avoid the issues of data scarcity and data imbalances. The performance of the best model was compared to three different balanced datasets of Databiox (i.e., 1200, 1400, and 1600 images) to determine the implications of this data augmentation. Furthermore, the effects of the number of epochs were analysed to ensure the coherency of the most optimal model. The experimental results analysis revealed that the proposed ensemble model outperformed the existing state-of-the-art techniques in relation to classifying the IDC-BC grades of the Databiox dataset. The proposed ensemble model of the CNNs achieved a 94% classification accuracy and attained a significant area under the ROC curves for grades 1, 2, and 3, i.e., 96%, 94%, and 96%, respectively.

## 1. Introduction

**Figure 1:**
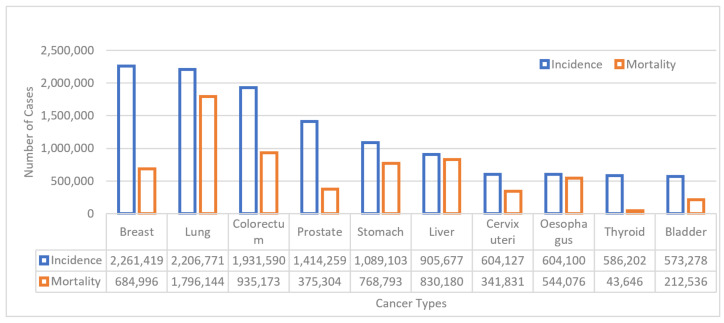
Estimated number of incident cancer cases and deaths worldwide due to BC.

Breast cancer (BC) is a prevalent type of cancer and has become the fifth leading cause of cancer-related deaths among women in recent years [1]. The most common signs and symptoms of BC include heaviness, stiffness, pain, and redness or swelling in the breast, as well as abnormalities such as shrinking, blood discharge, and nipple erosion [2]. Generally, cancer tissues originate either in the ducts or lobules of the breast and form a lump, which is referred to as a tumour. As per the position and nature of the mass of cancer tissues, the tumour is categorized into two types: Ductal Carcinoma In Situ (DCIS) and Invasive Ductal carcinoma (IDC). Invasive cancer spreads to other parts of the body, while in situ cancer does not invade other body parts [3]. Invasive cancer is life-threatening and 80% of BC deaths are caused by it. BC has been observed in younger age groups as well, and this incidence has been increasing rapidly. In India, a significant proportion of the younger population in their thirties and forties are affected by BC [4]. According to the International Association of Cancer Registries (IACR) and the Global Initiative for Cancer Registry Development, the BC incidence worldwide, in both sexes and all age groups, is depicted in Figure 1. In the year 2022, the estimated numbers of new cases and deaths in both females and males due to BC were 2,261,419 and 684,996, respectively [1].

The diagnosis of BC involves various screening methods, such as thermography, mammography, ultrasound scans, positron emission, magnetic resonance imaging, and breast-specific imaging [5]. Microscopy biopsies or surgical incisions are other gold standard methods used to diagnose the location of a lump and size of a tumour. However, the interpretation and analysis of BC types based on tissue textures, colour, and shape differentiation are challenging due to their similar clinical manifestations. Researchers are developing computer-aided diagnosis (CAD) systems based on artificial intelligence techniques to overcome such challenges [6]. CAD systems increase diagnostic accuracy by reducing human errors, which may further assist radiologists with proper diagnoses. Among the various artificial-intelligence-based techniques, deep neural networks are widely used in developing these CAD systems due to their automatic feature extraction, deeper network layers, and representation learning ability. In addition, advances in computer vision and the increased availability of computational power have contributed to the popularity of deep learning techniques. A vast amount of training data, more time, and high computational graphical processing units (GPU) are required to train these deep neural networks. However, a transfer learning approach can be used to overcome the requirement of a large amount of training data. Shallu Sharma and Rajesh Mehra performed a histopathological image classification using both transfer learning and training from scratch techniques [7]. They evaluated the performance of ResNet50, Xception, and Densenet121 pre-trained models as baseline models for feature extraction and achieved significant results.

In the line with this, an ensemble of pre-trained CNNs has been developed, which includes EfficientNetV2L [8], ResNet152V2 [9], and DenseNet201 [10] to extract the potential features for classifying the IDC-BC histopathological grades of a new dataset called databox [11]. The goal of this paper was to achieve a better performance in comparison to the state-of-the-art methods for breast IDC grade classification using histopathological images. The experimental work achieved the best results in comparison to the existing methods with the databiox dataset, to the best of the author’s knowledge.

**Contribution:**

- In the present study, an ensemble model comprising three pre-trained convolutional neural networks (CNNs) was designed to make grading predictions for the Databiox dataset, which consists of histopathological images of IDC-diagnosed patients for this grade classification. Different pre-trained base models were analysed for their performances individually and in combination to determine the most optimal and coherent solution for breast cancer grade classification.
- In Databiox, the dataset is imbalanced and the distribution of images among the different grades and total number of images in each grade are insufficient for training a CNN. This may lead the problem of bias towards one particular class with more images. To overcome this issue, data augmentation techniques were employed to balance the dataset. The performance of the best model was compared for three different balanced datasets of Databiox (i.e., 1200, 1400, and 1600 images) to ensure the limit of the data augmentation.
- Additionally, the implications of the number of epochs were also demonstrated throughout this experimental work. The performances of the models were observed for four different numbers of epochs, which further determined the robustness and coherency of the proposed ensemble model. The performance of the proposed models was analysed by utilizing the evaluation parameters, namely, precision, recall, f1 score, accuracy, ROC curve, and the area under the ROC curve (AUC).

## 2. Material and Method Used

In this work, CNNs were used as potential feature extractors and a fully connected neural network was used as a classifier. The performances of the CNNs were enhanced to extract the features effectively and the extracted features were mapped to their corresponding categories using the dense layers of the CNNs [7,8,9,10,12].

### 2.1. Dataset

**Figure 2:**
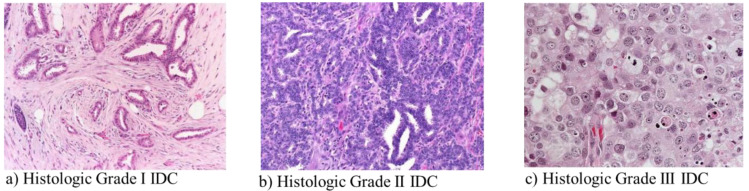
Histologic classification of IDC-BC grades with magnification factor 40×.

Here, histopathological images were analysed to classify the IDC BC grades. Different grading systems are available to determine the stage of cancer based on its tissue features, such as tubular formation, mitotic count (mitotic rate), and nuclear pleomorphism [13]. Each of these features was scored from 1–3 and the scores were added to obtain the final total score, which determined the grade. Grades 1, 2, and 3 correspond to total scores of 3–5, 6–7, and 8–9, respectively [14]. IDC is classified into three grades, namely low (grade 1), moderate (grade 2), and severe (grade 3). Figure 2 visually represents these different IDC-BC grades.

The classification of the different grades of IDC-BC using histopathological images is a challenging task due to the undifferentiable variants in these tissue images. To address this challenge, a robust and accurate grade classification model needs to be developed. The Databiox dataset provides such a platform, consisting of three grades with different magnification factors (4×, 10×, 20×, and 40×) collected from the Poursina Hakim research centre of the Isfahan University of Medical Sciences in Iran [11]. The dataset comprises histopathological microscopy images from 124 patients. It includes 259 images for grade 1, 366 for grade 2, and 297 for grade 3 BC, collected from 37, 43, and 44 patients, respectively, with a total of 922 images at four different magnification levels. All the images are in RGB colour and JPEG format, with resolutions of 1276 × 956 and 2100 × 1574 pixels.

### 2.2. Data Pre-Processing and Augmentation

In Databiox, the dataset is imbalanced and the distribution of the images among the different grades and total number of images in each grade are insufficient for training a CNN. Training CNNs with imbalanced data leads to a biased classification. To overcome this issue, the raw data of 922 images were transformed into a more balanced dataset of 1200, 1400, and 1600 images using data augmentation techniques [15]. This not only avoided the bias problem for each class, but also reduced the data scarcity issues.

To expand the dataset, an augmenter was used in the machine learning library, which is essentially a collection of options for common image-editing tasks, including rotating, shearing, and cropping. It is always important to select the appropriate data augmentation techniques to avoid any discriminating features or details. A random number generator was used to select values between −5 to 10, forming an augmentation pipeline. This resulted in the creation of a distinct image for each image sent through the pipeline. The original 922 histopathological images from Databiox were increased to 1200, 1400, and 1600 images for this experimental work by rotating with a probability = 0.7, maximum left rotation = 10, and maximum right rotation = 10, and zooming with a probability = 0.3, minimum factor = 1.1, and maximum factor = 1.6. After the augmentation process, the produced dataset was split into training and validation with a ratio of 80% and 20%, respectively. During the training, the validation set was used to validate the performance of the model, as well as to control the overfitting problem.

A batch normalization procedure was applied to normalize the selected features. Normalization is a commonly used process in machine learning that aims to scale down the features to make the model training less sensitive to the scale of the data. This process helps the model to perform better and maintain its training stability. It is an essential step in designing a CAD system, as it normalizes the data samples to obtain more consistent data for further processing. Therefore, batch normalization was performed in the pre-processing stage, which changed all the sample attributes to a single scale in the range from 0 to 1.

### 2.3. Convolutional Neural Networks (CNNs)

CNNs have indeed become a popular choice for deep-learning-based CAD expert systems, owing to their ability to learn the spatial hierarchies of features from raw input data [16]. Adding more layers to a CNN can increase its capacity to learn complex features from data, potentially leading to a higher accuracy [17].

**Figure 3:**
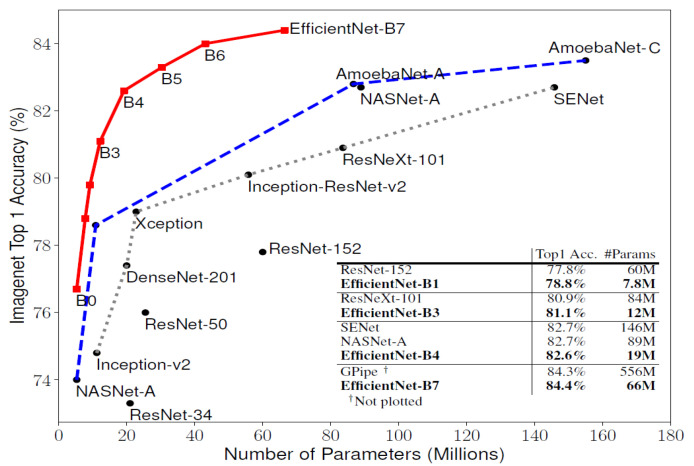
Comparative analysis of Top-1 accuracy and model sizes of different CNN models on ImageNet dataset, where EfficientNet outperforms other CNNs significantly [18].

Currently, various types of CNNs are available (Figure 3) and used as efficient feature extractors. Pre-trained CNNs are used to adopt a transfer learning approach when fewer computational resources are available [18,19]. Generally, transfer learning approaches make CNN models easy to use, particularly for task-specific classification [20]. Convolutional layers, pooling layers, and fully connected layers are the common types of layers in a CNN model, while different types of CNNs based on the arrangements of different layers, such as depth-wise separable convolution layers and skip connection between convolution blocks, have also been proposed [7,8,9,10]. One of the popular CNN architectures is ResNet, which uses a stack of linear, separable, depth-wise convolutional layers with residual connections [9]. One of the biggest problems in any deep learning network is vanishing and exploding gradients, which restricts us from going much deeper into the network. The core idea of ResNet is based on skip connections, which allow for activation to be taken from one layer and fed into a future layer, even if it is much deeper [21]. ResNet has achieved significant success in many computer vision tasks, including the ImageNet competition, where the Residual Network with a 152-layer variant (ResNet152) won in 2015 [9].

EfficientNet is a smaller and faster training system launched by Google AI that aims to provide a more efficient approach, as suggested by its name [8]. EfficientNet uses an efficient compound scaling mechanism, gradually increasing model attributes such as depth, width, and resolution, instead of arbitrarily scaling these parameters in the CNN design [18]. The recently introduced EfficientNetV2 improves upon EfficientNet in terms of its training time and parameter effectiveness [8]. DenseNet is another CNN architecture that requires fewer parameters than an equivalent traditional CNN, as it does not learn redundant feature maps [10]. Unlike ResNet, DenseNet concatenates the output feature maps of a layer with the incoming feature maps, instead of summing them up.

### 2.4. Ensemble of CNNs

**Figure 4:**
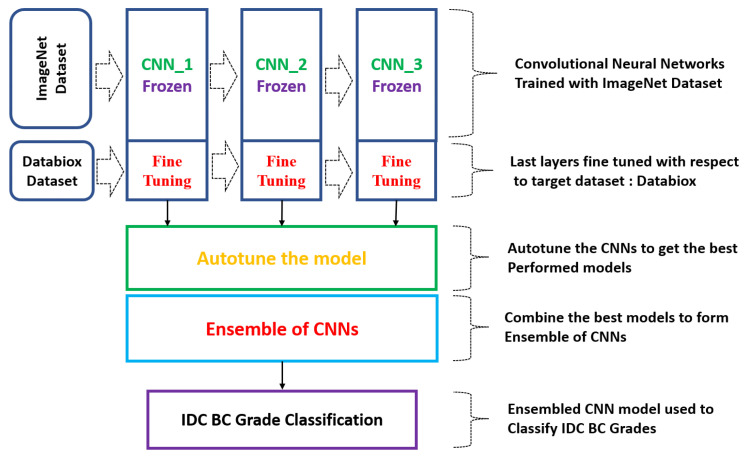
Methodology to classify IDC BC grades using Ensemble of CNN models.

This study aimed to compare the performances of individual CNNs with an ensemble of CNNs for the classification of IDC BC grades. The proposed model used an ensemble of CNN models to achieve a better performance. Initially, the CNN model was trained using the ImageNet dataset. Later, the pre-trained CNN model’s first few layers were frozen and the last layer’s output neurons were adjusted based on the number of classes in the target problem, as shown in Figure 4. The individual CNN model’s performance was observed and the best-performing individual models were saved for use in the ensemble architecture. Later, the best accuracy-providing models were combined to create the ensemble.

## 3. Results and Discussion

In this work, the IDC-BC grade classification task was performed on the Databiox dataset after applying pre-processing and data augmentation techniques. Three different augmented data samples were produced and used, consisting of 1200, 1400, and 1600 images from the original dataset of 922 images. For instance, in the case of 1200 histological images, 960 were used for training and 240 for validation. Similarly, for the 1400 histopathological images, 1120 were used for training and 280 for validation. Finally, for the 1600 images, 1280 were used for training and 320 for validation purposes. The data were split into an 80–20% ratio and the Google Co-lab platform was used to perform the experimental work. To evaluate the model’s performance, various metrics, such as the confusion matrix, precision, recall, F1 score, accuracy, receiver operating characteristic (ROC) curve, and area under the curve (AUC), were calculated using a python workflow.

### 3.1. Confusion Matrix

A confusion matrix was used to evaluate the performances of the models in the classification of the three IDC grades. Herein, the rows represent the predicted grades, while the columns represent the actual grades. Here, we discuss the confusion matrix of these IDC grades by considering a multi-classification that includes grade 0, grade 1, and grade 2 (Table 1).

A confusion matrix is an extremely useful performance metric for calculating recall, precision, specificity, accuracy, ROC, and AUC. The mathematical expressions used to calculate the precision, recall, F1 score, and accuracy were as follows:

Precision = TP/(TP + FP)(1)

Recall = TP/(TP + FN)(2)

F1 score = (2 ∗ Precision ∗ Recall)/(Precision + Recall)(3)

Accuracy = (TP + TN)/(TP + FP + TN + FN)(4)

A predicted positive grade that matched the actual positive grade was referred to as a true positive (TP). A predicted positive grade that did not match the actual positive grade was referred to as a false positive (FP). A predicted negative grade that matched the actual negative grade was referred to as a true negative (TN). A predicted negative grade that did not match the actual negative grade was referred to as a false negative (FN). For Grade 0, TP = GC00, FN = GC01 + GC02, FP = G10 + G20, and TN = G11 + G21 + G12 + G22. For Grade 1, TP = G11, FN = G10 + G12, FP = G01 + G21, and TN = G00 + G20 + G02 + G22. For Grade 2, TP = G22, FN = G20 + G21, FP = G02 + G12, and TN = G00 + G10 + G01 + G11. Table 2, Table 3 and Table 4 represent the experimental results produced by the individual models (via EfficientNetV2L, ResNet152V2, and DenseNet201) and the proposed ensemble of these CNN models. The performances of all the models were evaluated for three different data sample sizes (1200, 1400, and 1600) at four different numbers of epochs (5, 10, 15, and 20).

From Table 2, it was observed that the performances of the models improved when the number of epochs increased from 5 to 20. It showed that the models were trained well with a higher number of epochs. The performances of the individual models were not consistent; however, in comparison, the proposed ensemble model consistently achieved the best performance of all the models. Specifically, the proposed ensemble model achieved a maximum accuracy of 85%, a recall of 86%, an F1 score of 84%, and a precision of 87% for 20 epochs with a sample size of 1200. These results were significantly higher than the 72% accuracy obtained by Zaverah et al. using the same dataset [22].

Based on the results presented in Table 3, it was clear that the individual models did not exhibit consistent performances, but the ensemble of CNNs produced stable results. As the number of epochs increased from 5 to 20, the accuracy of the ensemble model also increased from 88% to 94% with a sample size of 1400. It is evident from the results that the representation learning ability of the model was enhanced with the large dataset. However, the sensitivities of all the models, except the ensemble model, were influenced towards one particular class when the sample size was increased from 1200 to 1400, eventually reducing their performances. These results significantly outperformed those achieved by Talpur et al. [23] and Sujatha et al. [24], who reported accuracies of 92.81% and 92.64%, respectively.

Furthermore, a similar trend was also observed in Table 4, where the performances of the individual models were not significant and inconsistent with 1600 samples. On the other hand, the proposed ensemble model still outperformed in comparison. The performance of the proposed ensemble model increased from 79% to 87% in terms of accuracy with an increment in the number of epochs, but decreased with the increment in the sample size from 1400 to 1600. The overfitting of the model with an increased sample size was the major rationale behind its degraded performance.

**Figure 5:**
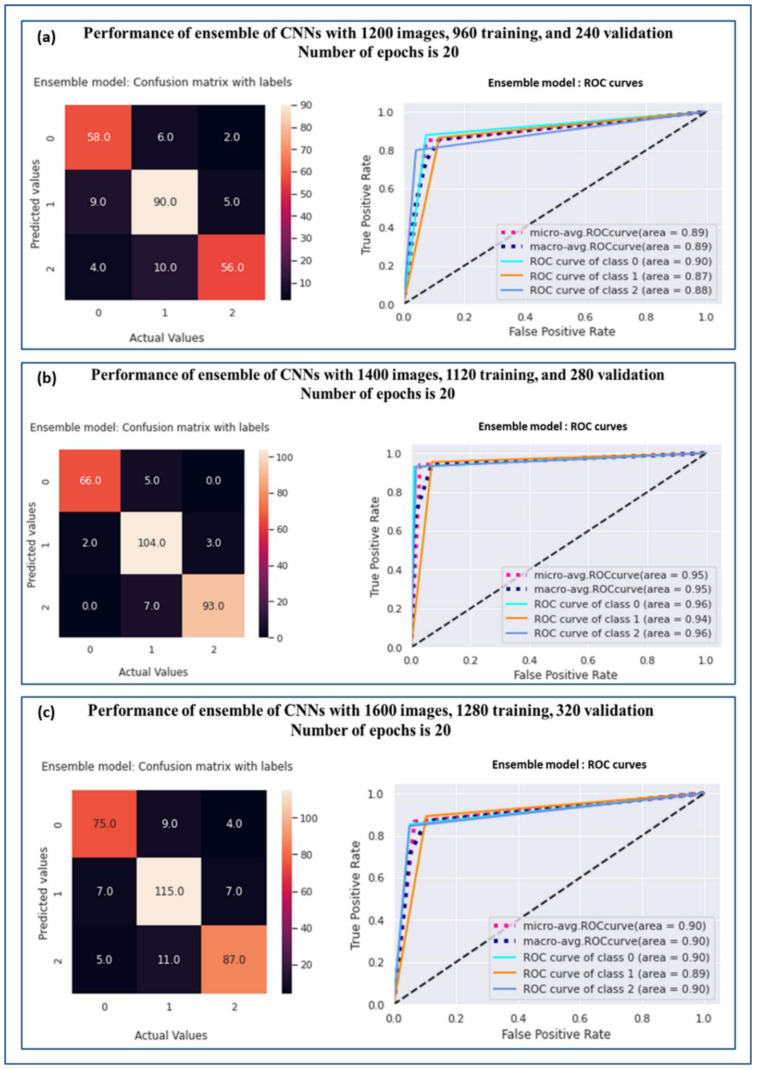
Confusion matrices and ROC curves for ensemble model over sample sizes (**a**) 1200, (**b**) 1400, and (**c**) 1600.

To obtain more insight into the models’ performance, confusion matrices and an ROC curve analysis were performed. Figure 5a–c illustrate the confusion matrix and ROC curve for the ensemble model over the sample sizes of 1200, 1400, and 1600, respectively. It was analysed from the confusion matrices in Figure 5a–c that the samples from grade 0 and grade 2 were wrongly predicted and were grade 1 in most cases. This was because the grade 1 stage is in between the stages of grade 0 and grade 2; therefore, they share some clinical expressions and this led to a state of confusion for the model. Despite this, the proposed ensemble model was overall the best with a sample size of 1400 and AUCs of 0.96, 0.94, and 0.96 for grades 0, 1, and 2, respectively (refer to Table 5).

#### Time Complexity

Time complexity is an important parameter that determines the time taken by an algorithm to execute each instruction of code. It was observed from the analysis that the computation time of the proposed model was positively correlated with the number of epochs and the sample sizes, as shown in Table 6. The time complexity increased with increments in the numbers of samples and epochs.

### 3.2. Comparison of the State-of-the-Art Techniques

This experimental work revealed the potential of data augmentation and an ensemble of CNNs for IDC BC grade classification. The performance of the proposed model was evaluated using the augmented dataset of Databiox with 1200, 1400, and 1600 histopathological images. In the case of the 1200 data size, the proposed ensemble model achieved a considerable performance with an accuracy of 85% and AUCs of 90% (grade 0), 87% (grade 1), and 88% (grade 2). Despite this significant performance, the model did not reach the existing state-of-the-art technique. This happened due to the scarcity of the data, and to obtain an improved performance of the system, we increased the data size to 1200, 1400, and 1600 by applying data augmentation techniques and implemented the same protocol. It was observed that the performance of the ensemble model drastically increased with the 1400 data size in comparison to the individual models. A remarkable result of a 94% accuracy was achieved with the proposed ensemble model. Zavareh et al. achieved an accuracy of 72% for grade classification using a transfer learning approach, Sujatha et al. achieved an accuracy of 92.64% by employing a transfer learning approach with DenseNet121, and Talpur et al. achieved an accuracy of 92.81% with the sequential CNNs. The proposed ensemble model surpassed the existing state-of-the-art techniques that have been reported using the Databiox dataset to date. A comparative analysis of the existing state-of-the-art techniques and the proposed ensemble model is illustrated in Table 7.

## 4. Conclusions

The present work proposed an ensemble model that improved the classification of IDC-BC grades and investigated the impacts of sample size and several training epochs. By implementing various data augmentation approaches, the sample size was increased and analysed for three samples sizes: 1200, 1400, and 1600. The obtained results revealed that there is always a trade-off between a model’s performance and the sample size. Training a model on a largely augmented dataset may introduce errors of generalization, which ultimately lead to overfitting problems. Therefore, datasets should be made sufficiently large through data augmentation techniques to ensure the optimal performance of a model. Furthermore, our study concluded that the ensemble of CNNs consistently achieved the most stable and robust performance in contrast to the individual models.

In the future, Generative Adversarial Networks (GAN) can be investigated to determine their overall impact on the accuracy of models in place of data augmentation. GANs have the potential to generate artificial data based on existing datasets. As a matter of fact, this will overcome the issues of data scarcity and data imbalances in the DataBiox dataset, which may increase the efficiency of the classification model.

## Figures and Tables

**Table 1:** A confusion matrix of IDC grades 0, 1, and 2.

Confusion Matrix		Predicted Class		
		Grade 0	Grade 1	Grade 2
**Actual Class**	**Grade 0**	GC00	GC01	GC02
	**Grade 1**	GC10	GC11	GC12
	**Grade 2**	GC20	GC21	GC22

**Table 2:** Classification report of CNN models (data augmentation performed and obtained 1200 images, of which 960 were training images and 240 were validation images).

Number of Epochs = 5					
CNN Model	Grade	Precision	Recall	F1-Score	Accuracy
Model 1: EfficientNetV2L	Grade 0	0.78	0.74	0.76	0.71
	Grade 1	0.72	0.62	0.67	
	Grade 2	0.66	0.79	0.72	
Model 2: ResNet152V2	Grade 0	0.35	0.82	0.49	0.45
	Grade 1	0.66	0.24	0.36	
	Grade 2	0.55	0.40	0.46	
Model 3: DenseNet201	Grade 0	0.87	0.33	0.48	0.47
	Grade 1	0.73	0.12	0.20	
	Grade 2	0.41	0.96	0.57	
Ensemble of CNN model (Model 1 + Model 2 + Model 3)	Grade 0	0.78	0.77	0.78	0.75
	Grade 1	0.67	0.82	0.74	
	Grade 2	0.85	0.65	0.73	
**Number of epochs = 10**					
**CNN model**	**Grade**	**Precision**	**Recall**	**F1-Score**	**Accuracy**
Model 1: EfficientNetV2L	Grade 0	0.70	0.87	0.77	0.76
	Grade 1	0.78	0.78	0.78	
	Grade 2	0.80	0.67	0.73	
Model 2: ResNet152V2	Grade 0	0.66	0.61	0.63	0.61
	Grade 1	0.65	0.53	0.58	
	Grade 2	0.55	0.69	0.61	
Model 3: DenseNet201	Grade 0	0.56	0.75	0.64	0.69
	Grade 1	0.77	0.57	0.66	
	Grade 2	0.75	0.78	0.76	
Ensemble of CNN model (Model 1 + Model 2 + Model 3)	Grade 0	0.78	0.80	0.79	0.78
	Grade 1	0.74	0.84	0.79	
	Grade 2	0.84	0.69	0.76	
**Number of epochs = 15**					
**CNN model**	**Grade**	**Precision**	**Recall**	**F1-Score**	**Accuracy**
Model 1: EfficientNetV2L	Grade 0	0.00	0.00	0.00	0.37
	Grade 1	0.38	0.83	0.52	
	Grade 2	0.34	0.16	0.22	
Model 2: ResNet152V2	Grade 0	0.28	1.00	0.43	0.28
	Grade 1	0.00	0.00	0.00	
	Grade 2	0.00	0.00	0.00	
Model 3: DenseNet201	Grade 0	0.00	0.00	0.00	0.34
	Grade 1	0.00	0.00	0.00	
	Grade 2	0.34	0.99	0.51	
Ensemble of CNN model (Model 1 + Model 2 + Model 3)	Grade 0	0.82	0.76	0.79	0.78
	Grade 1	0.76	0.80	0.78	
	Grade 2	0.78	0.78	0.78	
**Number of epochs = 20**					
**CNN model**	**Grade**	**Precision**	**Recall**	**F1-Score**	**Accuracy**
Model 1: EfficientNetV2L	Grade 0	0.74	0.83	0.79	0.82
	Grade 1	0.83	0.83	0.83	
	Grade 2	0.87	0.79	0.83	
Model 2: ResNet152V2	Grade 0	0.64	0.55	0.59	0.62
	Grade 1	0.66	0.72	0.69	
	Grade 2	0.55	0.56	0.55	
Model 3: DenseNet201	Grade 0	0.66	0.67	0.66	0.69
	Grade 1	0.66	0.80	0.72	
	Grade 2	0.83	0.56	0.67	
Ensemble of CNN model (Model 1 + Model 2 + Model 3)	Grade 0	0.82	0.88	0.85	0.85
	Grade 1	0.85	0.87	0.86	
	Grade 2	0.89	0.80	0.84	

**Table 3:** Classification report of CNN models (data augmentation performed and obtained 1400 images, of which 1120 were training images and 280 were validation images).

Number of Epochs = 5					
CNN Model	Grade	Precision	Recall	F1-Score	Accuracy
Model 1: EfficientNetV2L	Grade 0	0.64	0.94	0.76	0.79
	Grade 1	0.82	0.73	0.78	
	Grade 2	0.95	0.74	0.83	
Model 2: ResNet152V2	Grade 0	0.38	0.72	0.49	0.45
	Grade 1	0.51	0.38	0.43	
	Grade 2	0.52	0.33	0.40	
Model 3: DenseNet201	Grade 0	0.48	0.51	0.49	0.37
	Grade 1	0.26	0.11	0.15	
	Grade 2	0.35	0.56	0.43	
Ensemble of CNN model (Model 1 + Model 2 + Model 3)	Grade 0	0.89	0.80	0.84	0.81
	Grade 1	0.76	0.83	0.80	
	Grade 2	0.80	0.78	0.79	
**Number of epochs = 10**					
**CNN model**	**Grade**	**Precision**	**Recall**	**F1-Score**	**Accuracy**
Model 1: EfficientNetV2L	Grade 0	0.84	0.93	0.88	0.85
	Grade 1	0.88	0.80	0.84	
	Grade 2	0.84	0.86	0.85	
Model 2: ResNet152V2	Grade 0	0.94	0.21	0.34	0.53
	Grade 1	0.45	0.81	0.58	
	Grade 2	0.65	0.45	0.53	
Model 3: DenseNet201	Grade 0	0.54	0.77	0.64	0.62
	Grade 1	0.85	0.36	0.50	
	Grade 2	0.61	0.81	0.70	
Ensemble of CNN model (Model 1 + Model 2 + Model 3)	Grade 0	0.87	0.92	0.89	0.88
	Grade 1	0.86	0.84	0.85	
	Grade 2	0.90	0.88	0.89	
**Number of epochs = 15**					
**CNN model**	**Grade**	**Precision**	**Recall**	**F1-Score**	**Accuracy**
Model 1: EfficientNetV2L	Grade 0	0.96	0.93	0.94	0.88
	Grade 1	0.84	0.87	0.86	
	Grade 2	0.86	0.84	0.85	
Model 2: ResNet152V2	Grade 0	0.59	0.70	0.64	0.59
	Grade 1	0.67	0.45	0.54	
	Grade 2	0.53	0.65	0.59	
Model 3: DenseNet201	Grade 0	0.70	0.65	0.67	0.72
	Grade 1	0.65	0.84	0.74	
	Grade 2	0.86	0.63	0.73	
Ensemble of CNN model (Model 1 + Model 2 + Model 3)	Grade 0	0.87	0.94	0.91	0.91
	Grade 1	0.91	0.88	0.89	
	Grade 2	0.95	0.92	0.93	
**Number of epochs = 20**					
**CNN model**	**Grade**	**Precision**	**Recall**	**F1-Score**	**Accuracy**
Model 1: EfficientNetV2L	Grade 0	0.29	0.39	0.34	0.32
	Grade 1	0.35	0.24	0.28	
	Grade 2	0.33	0.36	0.34	
Model 2: ResNet152V2	Grade 0	0.00	0.00	0.00	0.36
	Grade 1	0.00	0.00	0.00	
	Grade 2	0.36	1.00	0.53	
Model 3: DenseNet201	Grade 0	0.23	0.58	0.33	0.27
	Grade 1	0.32	0.31	0.32	
	Grade 2	0.00	0.00	0.00	
**Ensemble of CNN model (Model 1 + Model 2 + Model 3)**	Grade 0	**0.97**	**0.93**	**0.95**	**0.94**
	Grade 1	**0.90**	**0.95**	**0.92**	
	Grade 2	**0.97**	**0.93**	**0.95**	

**Table 4:** Classification report of CNN models (data augmentation performed and obtained 1600 images, of which 1280 were training images and 320 were validation images).

Number of Epochs = 5					
CNN Model	Grade	Precision	Recall	F1-Score	Accuracy
Model 1: EfficientNetV2L	Grade 0	0.59	0.90	0.71	0.70
	Grade 1	0.92	0.50	0.64	
	Grade 2	0.68	0.80	0.73	
Model 2: ResNet152V2	Grade 0	0.31	0.90	0.47	0.37
	Grade 1	0.49	0.29	0.36	
	Grade 2	0.55	0.06	0.10	
Model 3: DenseNet201	IDC Grade 0	0.38	0.82	0.52	0.44
	Grade 1	0.45	0.10	0.16	
	Grade 2	0.53	0.60	0.56	
Ensemble of CNN model (Model 1 + Model 2 + Model 3)	Grade 0	0.78	0.72	0.75	0.79
	Grade 1	0.78	0.83	0.81	
	Grade 2	0.82	0.80	0.81	
**Number of epochs = 10**					
**CNN model**	**Grade**	**Precision**	**Recall**	**F1-Score**	**Accuracy**
Model 1: EfficientNetV2L	Grade 0	0.27	0.03	0.06	0.32
	Grade 1	0.39	0.09	0.15	
	Grade 2	0.32	0.85	0.46	
Model 2: ResNet152V2	Grade 0	0.00	0.00	0.00	0.40
	Grade 1	0.40	1.00	0.57	
	Grade 2	0.00	0.00	0.00	
Model 3: DenseNet201	Grade 0	0.00	0.00	0.00	0.38
	Grade 1	0.39	0.93	0.55	
	Grade 2	0.22	0.02	0.04	
Ensemble of CNN model (Model 1 + Model 2 + Model 3)	Grade 0	0.79	0.89	0.83	0.86
	Grade 1	0.92	0.84	0.87	
	Grade 2	0.86	0.86	0.86	
**Number of epochs = 15**					
**CNN model**	**Grade**	**Precision**	**Recall**	**F1-Score**	**Accuracy**
Model 1: EfficientNetV2L	Grade 0	0.36	0.04	0.07	0.34
	Grade 1	0.35	0.91	0.51	
	Grade 2	0.21	0.05	0.09	
Model 2: ResNet152V2	Grade 0	0.32	1.00	0.48	0.32
	Grade 1	0.00	0.00.	0.00	
	Grade 2	0.00	0.00	0.00	
Model 3: DenseNet201	Grade 0	0.26	0.56	0.36	0.25
	Grade 1	0.26	0.22	0.24	
	Grade 2	0.00	0.00	0.00	
Ensemble of CNN model (Model 1 + Model 2 + Model 3)	Grade 0	0.92	0.81	0.86	0.86
	Grade 1	0.81	0.94	0.87	
	Grade 2	0.88	0.83	0.85	
**Number of epochs = 20**					
**CNN model**	**Grade**	**Precision**	**Recall**	**F1-Score**	**Accuracy**
Model 1: EfficientNetV2L	Grade 0	0.24	0.64	0.35	0.24
	Grade 1	0.45	0.04	0.07	
	Grade 2	0.19	0.15	0.16	
Model 2: ResNet152V2	Grade 0	0.23	0.14	0.17	0.39
	Grade 1	0.42	0.88	0.57	
	Grade 2	0.00	0.00	0.00	
Model 3: DenseNet201	Grade 0	0.43	0.28	0.34	0.47
	Grade 1	0.48	0.67	0.56	
	Grade 2	0.48	0.39	0.43	
Ensemble of CNN model (Model 1 + Model 2 + Model 3)	Grade 0	0.86	0.85	0.86	0.87
	Grade 1	0.85	0.89	0.87	
	Grade 2	0.89	0.84	0.87	

**Table 5:** Comparative analysis of the proposed ensemble model performance for different sample sizes at 20 epochs.

Sample Size	Grade	Precision	Recall	F1-Score	AUC	Accuracy
1200	Grade 0	0.82	0.88	0.85	0.90	0.85
	Grade 1	0.85	0.87	0.86	0.87	
	Grade 2	0.89	0.80	0.84	0.88	
1400	Grade 0	0.97	0.93	0.95	0.96	**0.94**
	Grade 1	0.90	0.95	0.92	0.94	
	Grade 2	0.97	0.93	0.95	0.96	
1600	Grade 0	0.86	0.85	0.86	0.90	0.87
	Grade 1	0.85	0.89	0.87	0.89	
	Grade 2	0.89	0.84	0.87	0.90	

**Table 6:** Time complexity of the proposed ensemble model for different sample sizes and numbers of epochs.

Samples	Epochs	Time for Data Augmentation (In Minutes)	Time for Training and Validation (In Minutes)	Total Time (In Minutes)
1200	5	21	27	48
	10	21	33	54
	15	21	46	67
	20	21	50	71
1400	5	24	30	54
	10	24	39	63
	15	24	48	72
	20	24	53	77
1600	5	28	34	62
	10	28	42	70
	15	28	49	77
	20	28	58	86

**Table 7:** Performance comparison of the proposed ensemble model with the existing state-of-the-art techniques on the Databiox dataset for the classification of IDC-BC grade images.

Reference	Year	Approach	Performance Metric
Zavareh et al. [22]	2021	Transfer learning approach (VGG16 used as feature extractor)	Accuracy of 72%
Kumaraswamy et al. [25]	2021	Transfer learning approach pre-trained CNNs: DensNet201 and NASNetMobile used as feature extractors)	Accuracy of 72%. AUC for Grade 1, and Grade 2 is 98% and 75%, respectively with DensNet201 AUC for Grade 3 is 69% with NASNetMobile
Sujatha et al. [24]	2022	Transfer learning approaches (Utilized VGG16, VGG19, InceptionReNetV2, DenseNet121, and DenseNet201)	DenseNet121 produced the highest accuracy of 92.64%
Talpur et al. [23]	2022	A sequential convolutional neural network is utilised	Accuracy of 92.81%
Present Work	2023	Proposed Ensemble Model(EfficientNetV2L + ResNet152V2 + DensNet201)	Accuracy of 94%. AUC of 96%, 94% and 96% for Grades 0, 1, and 2, respectively.

## Data Availability

We haven’t generated any new data; however, the data set we utilized is publicly available at https://databiox.com/datasets/. Interested individuals can register and retrieve the same for research purposes.
